# Social Image of Old Age, Gendered Ageism and Inclusive Places: Older People in the Media

**DOI:** 10.3390/ijerph192417031

**Published:** 2022-12-18

**Authors:** María Sánchez-Román, Gadea Autric-Tamayo, Gloria Fernandez-Mayoralas, Fermina Rojo-Perez, María Silveria Agulló-Tomás, Diego Sánchez-González, Vicente Rodriguez-Rodriguez

**Affiliations:** 1 Grupo de Investigacion sobre Envejecimiento (GIE), IEGD, CSIC, 28037 Madrid, Spain; 2 University Institute on Gender Studies and Social Analysis Department, University Carlos III of Madrid, Getafe, 28903 Madrid, Spain; 3 Department of Geography, National Distance Education University (UNED), 28040 Madrid, Spain

**Keywords:** gendered ageism, active ageing, age-friendly communities, ageing in place, gender, media, wise words

## Abstract

Ageism promotes the exclusion of older people from society by generating a negative image that they also internalize. The aim of this article is to investigate older people’s social self-image, through statements broadcast on a national Spanish radio program aimed at this group. A qualitative analysis was conducted for a random sample from the sound archive for the Radio Nacional de España program Juntos Paso a Paso (Together, Step by Step) (2008–2021), using codes based on the pillars and determinants of active ageing and the three dimensions of ageism. Intercoder agreement was calculated. There were significant findings regarding ageism, gendered ageism and ageing in place, with differences according to size of municipal area. The program in question can be considered a viable secondary source for the research aim. Ageism is most commonly manifested through implicit opinions and invisibilization in family and social contexts. Care activities play a notably central role in responses related to gendered ageism. In relation to ageing in place, older people prefer their habitual environment when they have moderate care needs and accept moves to nursing homes when their needs increase.

## 1. Introduction

Health, participation and lifelong learning, and security are the three pillars of active ageing established by the World Health Organization’s policy framework [1], a multidimensional tool oriented to enhance quality of life as people age. The framework also identifies a series of determinants, with two cross-cutting determinants (gender and culture) in addition to personal, behavioral, economic, health and social services, physical environment and social determinants. In 2015, the WHO also defined healthy ageing to promote wellbeing in older age, allocating a fundamental role to the opportunity to live in environments that support and maintain intrinsic capacity and functional ability. The environments covered in this model include the home, the community and the wider society, with multidimensional factors that encompass concepts based on an interpretation of ageing as a lifelong process, such as the built environment, attitudes and values, health and social policies, support systems and services implemented by those systems [2]. These concepts are based on an interpretation of ageing as a lifelong process.

Following both premises, the WHO implemented the age-friendly cities and communities programme [3] and subsequently, the plan for a Decade of Healthy Ageing (2020–2030) in connection with the Sustainable Development Goals. Age-friendly cities (and communities) encourage active ageing by making structures and services accessible and inclusive for older people with varying needs and abilities [3]. The Decade of Healthy Ageing was proposed to provide opportunities to develop integrated healthcare and social services and create environments that are adapted and favourable to older people in communities, cities and countries, extending the network of age-friendly cities and communities [4]. Active ageing in age-friendly environments supports and promotes growing older at home, with autonomy and independence to develop residential strategies based on the residential wishes and living conditions of older people [5]. The WHO’s promotion of active and healthy ageing represented a rights-based approach, and the guiding principles of the Decade of Healthy Ageing supplemented the 2030 Agenda in addition to the principles inspiring the global strategy and campaign to combat age-based discrimination and ageism [4]. The active ageing concept interpreted this rights-based approach as a change that “recognizes the rights of people to equality of opportunity and treatment in all aspects of life as they grow older” [1]. This implies the inclusion of a gender perspective (Ramos Toro, 2017), in addition to a broader intersectional perspective [6] that takes other dimensions (age, ethnicity, culture, religion and key sociodemographic variables) into account.

Age-based discrimination is harmful to health and wellbeing, as it is a significant barrier to the enacting of effective healthy ageing policies and measures related, as recognized by WHO Member States in the global strategy and action plan on ageing and health and through the Decade of Healthy Ageing: 2021–2030 [7].

Ageism, defined as a range of stereotyped ideas and images that cause prejudice and discrimination against a group based on its age [8], poses an obstacle to active ageing [9] insofar as it promotes the exclusion of older people from society [10] and also encourages older people to internalize ageist ideas and self-exclude [11]. Older people construct an image of old age and ageing that cannot be separated from the place in which they are ageing, based on their living environment, social participation and the availability of inclusive spaces [12]. This can also vary according to the rural or urban nature of a habitat, the cultural context and the social setting [13]. It can be expressed in seemingly arbitrary fashion [14] or be related to mediators that have differing impacts on social exclusion [15]. In this regard, the WHO’s age-friendly communities and cities framework encourages a shift in attitudes, behaviours and messages towards older people, focusing on inclusion and respect [3].

From this inclusive perspective, the WHO has also warned about “gendered ageism”: gender-related differences in the ageism that men and women face, with emphasis placed on the greater impact of ageism on older women [7]. Older women tend to suffer ageism in the workplace, which affects their professional opportunities and hence their pensions [16]. Women are also required to assume a dual role during their lives, bearing responsibility for household tasks and as employees in the workforce. They face unfair policies such as losing pension contributions during maternity leave and inequality of opportunity in the workplace as they age [17]. All of this can affect their future health and independence. Gendered ageism is thus a social determinant of health, and older women are negatively affected due to both their age and gender, with significant consequences in terms of wellbeing [18]. Gendered ageism is also institutionalized in healthcare practices, especially in relation to caregiving due to the role women play as primary caregivers and because they may need more long-term care services than men [19].

The WHO active ageing framework also describes gender as a “lens” through which to consider the development and application of public policies [1]. There is recognition of the disadvantaged position that women face as their lives unfold, with a greater or lesser impact depending on the country, across all key areas (education, access to healthcare, nutrition, work and caregiving work, and inheritance). This framework introduced the life course perspective, stressing the need to take into account inequalities caused by gender roles and stereotypes over a lifetime in order to secure a full old age. From a healthy ageing perspective, gender also significantly influences inequality and gives rise to very different ageing pathways, particularly among older women [4].

The media can contribute to ageism (and sexism) by shaping a single social image of old age that is far from the diverse and plural modern reality [20], while social media provides an online platform for the reproduction and reinforcement of the ageist (and sexist) tropes present in traditional media [21]. Amundsen [22] highlights the consensus in the literature regarding the influence of media on cultural attitudes, reflecting norms of social acceptability regarding ageing in the construction of age-related discourses. Ongoing age-based discrimination in the media reflects cultural norms and beliefs that are considered socially acceptable. Unfortunately, the presence of ageist images and discourses intensified during the pandemic, both in traditional media [23] and on social media [24].

However, the media can also encourage the construction of social reality from a perspective of heterogeneity of ageing and old age [25]. As stated by Suay Madrid [26], this group has not received proper attention in terms of communication ethics. There is a need for a new outlook involving more appropriate treatment and emphasising service-based, committed journalism that encourages the construction of a more inclusive society. This society should involve active participation and involvement from older people as co-protagonists in the information process, transcending the view of information as a “goal” in and of itself and making it a “medium”—a personal tool for the target audience.

In this regard, numerous studies have researched visual ageism as it affects older people in the media [27], but there is scarce research taking older people into account as protagonists who construct their image, undoubtedly influenced by society.

The aim of this article is to explore and analyse the opinions and experiences of older people reflected in the radio programme Juntos Paso a Paso (Together, Step by Step) between 2008–2021 from the framework of the three dimensions of ageism (discrimination, stereotypes, and prejudices) and the pillars and determinants of active ageing. This analysis will consider other dimensions outlined in the literature as relevant factors in the quality of life of older people, such as gender and place of residence.

## 2. Materials and Methods

The research used the sound archive for the Radio Nacional de España (RNE) programme Juntos Paso a Paso (Together, Step by Step) from when it started to be broadcast in October 2008 until December 2021. The accessed data has been used according to the agreement signed between the corporation Radio Televisión Española (RTVE) and state agency Spanish National Research Council (CSIC). The programme is aimed at listeners aged 65 years and above. Its website (https://www.rtve.es/play/audios/juntos-paso-a-paso/, accessed on 13 April 2022) lists content related to travel, active ageing, adapted sport, healthy living, literature, university for mature students and workplace integration. It is stressed that older people are the protagonists; they participate each weekend with visits made to seniors’ recreation centers, nursing homes and special employment centers, as well as by leaving comments, requests, poems, and songs with an automatic answering service. Listeners are also looking for information, entertainment, and company, but the intention is to offer a different perspective, “full of fight and hope; a demanding vision”, as the website states.

The programme presents the aforementioned content through expert interviews. Other sections directly involve the participation of older people. These include the “answerphone” segment, containing recorded messages sent into the programme, and the segment Wise Words (“Palabras Mayores”), involving interviews with older people living in various geographical and residential contexts throughout Spain.

The programme was analysed by classifying each episode by date, location and summary, including size of municipality (fewer than 10,000 inhabitants; 10,000 to 49,999 inhabitants; 50,000 to 499,999 inhabitants; and 500,000 or more inhabitants) (Table 1) and physical/social daily environment (including seniors’ recreation centers, nursing homes, and universities) (Table 2).

The preliminary part of the classification process involved listening to the segment of interest for this article, Wise Words, in order to prepare research notes that could be used for the subsequent analysis. The average duration of this segment was five minutes.

A total of 640 programs were classified, of which 542 included the Wise Words segment. A sample of four programs per year was selected on a random basis, producing a total of 56 documents that represented a little over 10% of potential transcripts. In addition, and to secure representation for the relevant stratum, the classification process identified programs produced with a municipal population of fewer than 10,000 inhabitants (N = 11). One such program was already part of the randomly selected sample (Table 1).

The research team then transcribed the segments, and the classified information and transcripts were moved to a master project to be analysed using the ATLAS.ti (V9) qualitative analysis software. This master project was distributed among the team.

Given the main aim, the team decided to carry out a qualitative content analysis with the total number of selected documents (*n* = 66). From its beginnings at the start of the twentieth century, content analysis was based on the use of a more quantitative approach to the study of communication: on quantifying material analysed via the frequency of use of significant words of terms [28]. However, the use of content analysis from a qualitative perspective goes beyond quantification. It is intended to examine the features that older adults define in terms of the social image of old age, as a group belonging to society as a whole [28,29,30]. In other words, as explained by Schreier [31], for this paper it was understood that “qualitative content analysis is a method for systematically describing the meaning of qualitative data”. Hence, the main purpose of applying qualitative content analysis in this work was to produce a valid and replicable categorization of the content appearing in the statements and unveil the underlying issues that give meaning to the social image of ageing for older people [30,32,33].

This search for a deeper meaning is precisely what characterizes the use of a qualitative methodology in this work, rather than a mere amassing of data with the danger of creating a statistically limited (though mathematically correct) picture of the subject at hand [34].

Two theories tend to underpin quantitative and qualitative media research [35]. One, cultivation theory, proposes that media messages can cause heavy television users to see the world through the lens of the messages broadcast on television. The other, social cognitive theory, argues that the impact involves the recipient of the messages but also their resemblance to the actor or broadcaster, and the positive or negative consequences of the broadcast acts. This means that when studying the social image of old age, it is useful to examine the media messages broadcast by older adults, which reach a group of listeners that includes older adults themselves.

Additionally, content analysis is a technique that has been closely linked to media research since its beginnings [29,36]. Specifically, press and radio analysis attracted the most initial interest, though television soon overtook radio and partly eclipsed press [37]. Nonetheless, a large proportion of the Spanish population uses the radio, with over 22 million daily listeners in the most recently analysed period [38]. The latest audience figure for the radio program subject to analysis in this study (Juntos Paso a Paso) was 130,000 listeners, which reached 230,000 during the pandemic. Moreover, 68.5% of listeners were aged 65 and above (data provided by the media themselves, who did not distribute the report upon request).

The research team carried out simultaneous blind coding in two sub-teams, using a code book with defined meanings that had been previously created based on the pillars and determinants for the active ageing framework [1] and the WHO definition of the concept of ageism [7]. Using previously created codes and the simultaneous blind coding method made it possible to calculate intercoder agreement (ICA) [33] and Krippendorff’s Cu-alpha.

The team decided to use Krippendorff’s Cu-alpha in view of several aspects, including the number of simultaneous coders (four) and this statistic’s reliability in terms of ruling out errors or external effects, as noted in reviews by other authors [39]. In terms of reliability, other widely-used statistics such as Cohen’s kappa have been discarded for presenting certain limitations regarding non-coding aspects. As stated by Zhao [40], “Cohen’s kappa is not a general measure for inter-rater reliability but a measure of reliability that only holds under particular conditions, which are rarely met”. Although there is no broad consensus on which statistic is best to use, Krippendorff’s Cu-alpha is one of the most widely used in media research in recent years, as well as one of the most reliable when there are more than two coders and small samples [41]. Although the sample used in this paper contained a large number of documents (66 in total), the material was short in length. Finally, Krippendorff’s Cu-alpha had been adapted and revised for use in the software used by the team for this work, ATLAS.ti (V9) [42].

Lower-than-desirable levels were ultimately obtained (Krippendorff’s Cu-alpha = 0.3 for the total range of codes). However, the level of agreement increased for certain codes used in the post-calculation discussion, coinciding with those that gave the analysis higher qualitative significance (Krippendorff’s Cu-alpha = 0.5). Moreover, several causes for this situation were identified and are explained in detail in the study limitations section. In addition to the previously created codes, the team agreed to create new codes in case they might be required owing to the content of the documents.

## 3. Results

Out of the total number of programs, a higher prevalence of content was found relating to disability among older people between 2008 and 2013, with 2009 containing the highest number of references to this topic (19 times, 25.3% of the total).

The geographical distribution of the programs showed a dominance for the Community of Madrid over other Autonomous Communities (414 of the 542 programs classified, or 76.4%). In terms of size of municipality, a large majority of programs were located in municipalities with populations of 500,000 or more (65.5% of the total), with 19.9% in municipalities with 50,000 to 499,999 inhabitants, 6.1% in municipalities with 10,000 to 49,999 inhabitants, and only 2% in municipalities with fewer than 10,000 inhabitants. The remaining programs were either located in several municipalities of varying sizes or did not identify the municipality (6.5%).

There was a degree of diversity representation in terms of the daily environment experienced by those interviewed in the “wise words” segment, although most interviews took place in seniors’ recreation centers (51.1%) and in nursing homes (27.5%). Others came from contexts including day care centers for elderly people (6.8%), calls made to the program (3.9%), and cities and villages that were visited for purposes of interviews (1.8% and 1.5%, respectively). The remaining percentages corresponded to interviews with older members of academic institutions (1.7%), older participants in authorities, associations, federations, foundations and organizations (5.2%), and unidentified contexts (0.6%).

The thematic content obtained from the random sampling documents (N = 56) and the other 10 documents located in municipalities with fewer than 10,000 inhabitants featured various qualitatively significant topics (Table 3), which can be classified in three thematic areas:

- References to ageism, particularly implicit references or references to the invisibilization of older people.
- Links between gender and the most prominent areas of life featured in the responses (family network, caregiving, use of time, social participation).
- Opinions on social and healthcare services and the setting in which ageing takes place (growing older at home).

Literal quotations are identified by document number (D#), program year of broadcast, sex of speaker, residential setting (see Table 2) and geographical context (urban or rural). Square brackets ([]) indicate content or comments omitted from the transcript.

### 3.1. Implicit and Latent Ageism: The Invisibilization of Older People

Most ageism-related topics appeared through implicit expressions, meaning manifestations of age-based stereotypes, prejudices, or discrimination in the form of subconscious or unintentional thoughts, emotions or actions. They also arose in relation to the invisibilization of older people, with mentions of obstacles blocking older people’s involvement and protagonism in decisions regarding their health, social participation, way of life, use of time and financial resources.

In both cases, references to ageism were closely related to social determinants associated with the family network (the most frequently mentioned code in the entire analysis) and other gender-related codes, particularly the care given by older people to their family members.

There were clearer expressions of ageism in responses from urban areas (with populations in excess of 10,000 people). These topics appeared sporadically in rural areas, to note the essential and not always socially recognized role that older people play in their families by caring for grandchildren or the provision of financial support in times of crisis:

*“Total paralysis for everyone who goes to work and has small children, you know. […] I think it’s the best thing grandparents can do: take care of the children.”*

(D7, 2009, woman, municipality, rural)

*“It would be a disaster, a disaster. Because they couldn’t go to work, they couldn’t do anything if we didn’t help out.”*

(D7, 2009, woman, municipality, rural)

*“I’ve brought up my grandkids. If it hadn’t been for us, my children couldn’t have worked.”*

(D7, 2009, man, municipality, rural)

*“In a financial sense, I think it’s absolutely crucial at the moment. And the fact that only some of us can, I think we’re holding back and we’re not doing what we want because we’ve got people behind us who we need to support a bit. Because for me, they’ve got it a fair bit worse than us.”*

(D61, 2012, man, municipality, rural)

This issue also appeared in urban municipalities, although older people placed more emphasis on their excessive caregiving burden, including describing it as an excess imposed by their children that did not benefit the older people or their grandchildren. This was identified by the men:

*“At an earlier stage, the truth is that we were babysitter-grandparents. We were there every day, taking the place of a nursery.”*

(D25, 2014, man, seniors’ recreation center, urban)

*“I just think that sometimes our children take advantage, and other times it’s a necessity.”*

(D25, 2014, man, seniors’ recreation center, urban)

*“I’m against babysitter-grandparents, because it’s no good for the grandparent and it’s no good for the child. I mean, grandparents should be there when needed, once in a while, but I don’t think it’s good for a kid to be constantly with an older person. Kids have to be with other kids and grandparents should be there for exceptional cases. […] Creating dependence where your life is limited because you’ve got the kid, it’s no good for the grandparent and it’s no good for the kid either.”*

(D25, 2014, man, seniors’ recreation center, urban)

Meanwhile, the women focused more on the inconvenience of the burden insofar as it restricted their freedom in terms of their leisure time:

*“I don’t do babysitting, but I do think it’s taking advantage sometimes. I have a close friend who looks after all of her children’s kids and she’s exhausted, and on top of that I’m always seeing her with shopping trolleys, shopping and shopping. Of course, you’ve got to feed all the kids and the mothers as well, they come for lunch too. And she says—just to me, you know– that she’s tired and that during this time she could be out somewhere, taking a trip, doing something.”*

(D25, 2014, woman, seniors’ recreation center, urban)

*“Older people have a limit too, so naturally it’s true that they take advantage a bit, and sometimes it’s not so good, either. Older people need their own space, their freedom…”*

(D49, 2020, woman, seniors’ recreation center, urban)

On other occasions, older people described the restrictions placed on their decision-making capacity in situations involving their children divorcing, as well as the resulting loss of their rights as grandparents. As the following quote from a female senior recreation center user indicates, this is a loss for both grandparents and grandchildren:

*“Grandparents have the same rights as parents, because if they’re your blood, the parents’ rights end where the grandparents’ rights to see the children start, and you have to fight for that. […] There’s no reason to stop children from seeing their other family members. And most of all, their grandparents.”*

(D13, 2011, woman, seniors’ recreation center, urban)

Stereotyped opinions on younger and older people appeared in both rural and urban environments, regarding the contrasting habits of both generations and aspects affecting their relationship. This was particularly notable in the form of comments regarding street parties:

*“These days, if they’re not drinking in the streets or whatever then they’re not happy. And if I don’t give them money they get mad, and if you say anything then everyone else is an old fogey. You know, there’s a big difference in life nowadays.”*

(D58, 2010, man, nursing home, rural)

References to digital habits as a barrier to intergenerational communication were more common in urban contexts, where the continuous use of electronic devices by younger people compared to their grandparents caused a sense of a communication gap when the generations were together:

*“My grandson is in his room at ten in the morning, bang bang bang, and he spends hours slumped at the computer, downloading films, or music, or this, or that, it’s a nightmare.”*

(D2, 2008, man, seniors’ recreation center, urban)

*“They’re using their little machines [pejorative], ping ping ping… and they’re on it for an hour, and it’s like you weren’t even there.”*

(D21, 2013, man, seniors’ recreation center, urban)

*“They’re in their world and the fact is you can hardly ever have a conversation.”*

(D21, 2013, man, seniors’ recreation center, urban)

*“Everything sounds foreign. They have to explain all these slang words they’re making up, or not making up, but lots of the time we don’t know… the definitions, what they mean. Or we have to be asking: so what does it mean? And what’s this? And of course we give up lots of the time, because we say well, this isn’t for us.”*

(D17, 2012, woman, seniors’ recreation center, urban)

This notion extended to habits during the COVID-19 pandemic, with young people blamed for spreading the virus:

*“Young people are going to have to stop a bit, because it’s them spreading the pandemic, they’re spreading it to the family, aren’t they? These parties they’re having at night… and the way of life that’s being forced because of how irresponsibly most young people are acting.”*

(D51, 2020, man, nursing home, urban)

Finally, references were made—particularly in urban settings—to the meaning of the social representation of old age. Older people were portrayed as not important to contemporary society nowadays, and hence as an ignored group. Speakers felt this was also reflected in the media:

*“I have the feeling that old people aren’t in fashion, and so they’re ignored. […] It’s the culture of beauty, it’s not the culture of old people, we’re not in fashion.”*

(D35, man, foundation, urban)

*“I think that the image that’s portrayed of older people in the media: normally it’s when something happens and it’s usually almost all negative. […] So no, I honestly think that older people, not just because we’re older but because together we’ve accumulated many years of experience and wisdom and lots of patience […] I think they should rely on us and we should be represented at lots of institutions and on lots of official bodies.”*

(D35, 2016, woman, foundation, urban)

*“We’re living in a disposable age: if it doesn’t work, it’s thrown away. With furniture and everything … household appliances, ten years; cars, ten years. So with all due respect, older people are marginalized in the family, because it can’t be any other way. It just can’t be any other way.”*

(D5, 2009, woman, seniors’ recreation center, urban)

There were also references to how old age is construed by some older people, especially in reference to the portrayal of seniors’ recreation centers as places for “old codgers”, when in reality the resources directed at this group are generally valued by their users

*“You tell lots of people: ‘Hey, I go to a centre, come along,” and so on, because they’ve retired. And they don’t say “older people” they say: ‘Oh! An old folk’s centre, I’m not going to an old folk’s centre.’ I mean, a seniors’ recreation center, even if they’re old, but they’re there. They come along, read the newspaper, get along with people and the truth is that everyone who comes along changes for the better, straight away.”*

(D67, 2009, woman, seniors’ recreation center, urban)

*“Lots of people tell me: “no, no, I’m not retiring because I won’t know what to do, I’ve got my job and I’m going to do it, I’m not going to do nothing. If I’m going to end up just doing nothing, I’ll stay where I am, working”. […] Well if they don’t retire I think that’s why, because they say ‘what will I do then, I’ll keep working here.’ They don’t come to these places or find out what it’s like. Because I’ve got, I’ve got a brother-in-law who says, of course, he’s not going to these centres because only old folk come here.”*

(D26, 2014, man, seniors’ recreation center, urban)

*“When I retired, I said: ‘where will I go?’ I felt like I could still be useful and there was still plenty I could do. And I thought about it and I was alone and I felt… really bad. So I said: ‘fine, I’ll go and see the retired people here.’ And I came here, I met all the people like the social workers, the manager, all the people who work here: fabulous, marvellous. It’s marvellous because they really encourage you, they pick you up and they show you the way a bit.”*

(D67, 2009, woman, seniors’ recreation center, urban)

### 3.2. Gendered Ageism, Family Network and Use of Time

Gender topics were essentially related to the use of time, family network, caregiving, occurrences of implicit sexism and social participation.

There was a notable difference between men and women in terms of forms of socialising and use of time in rural municipalities. This was exemplified by the statements made by participants regarding the impact of the 2008 economic crisis on their environment. Women commented more on domestic activities, such as buying household supplies, while men referred to changes in the behaviour of their peers in public spaces such as bars:

*“You go out to buy anything, it’s all much more expensive, and our pensions aren’t going up.”*

(D61, 2012, woman, municipality, rural)

*“You can tell in every part of daily life, because it’ll be on the street. I’m one of those who likes to have my little drinks at midday, and in the evenings if I can. And I’ve noticed that I think I have less, and I think people around me are having less, because while there used to be fifteen or twenty or twenty-five of us meeting up, meeting up there every day for a couple of glasses of wine, now there are ten of us, or eight, or fewer.”*

(D61, 2012, man, municipality, rural)

These gender differences are also observed through the preferred choice of certain kinds of tourism, such as spa tourism, an option for women more than for men:

*“I’ve been to all the spa resorts, but only taking people and collecting them. That’s when I was working and we took people from here to Portugal, for example. […] when I retired, I didn’t miss a single IMSERSO trip. As soon as I got back from one I was signing up for another, so that’s why I’m telling you I’m going to do all the ones I wanted to. However, I haven’t been to any spa resorts.”*

(D65, 2014, man, nursing home, rural)

These same rural municipalities feature life pathways that are clearly defined by sex-based occupational segregation: housework for women and driving or security jobs for men:

*“Like the ID card says, “housework”. I was the oldest of six siblings.”*

(D66, 2014, woman, nursing home, rural)

*“I worked in a porcelain factory, making China crockery and figures, we called it porcelain or something like that.”*

(D66, 2014, woman, nursing home, rural)

*“At the beginning, a lorry driver. Then, buses, that was more being with people and more relaxed. I was a driver and I’ve been a driver for more than forty years right here.”*

(D66, 2014, man, nursing home, rural)

*“The last job was a chief. Well, until 88 when I had the problem with my arm and I had to leave the kitchen. And then I worked for the Community of Madrid, from 88 onwards, at an exhibition centre. And before then, I worked from the age of seven. I did everything except for the nice jobs [laughing].”*

(D66, 2014, man, nursing home, rural)

Issues related to family networks largely concerned caregiving, particularly in terms of grandchildren. Contributors described the satisfaction involved in being able to interact with their grandchildren but also the invisible and general overburdening referred to in previous quotations. They also referred to caring for their partners. There was more emphasis placed on willingness to care for grandchildren in rural areas:

*“Well, I’m always there, I’m there. I have a grandson who’s the best thing in my life, and I’m there and I will be for as long as I can. Can I offer a hand? Yes, I can offer a hand, of course. You can always do something, whether it’s big or small. […] For as long as I’m alive, my grandson won’t lack for anything from me; I’m sure of that.”*

(D62, 2012, woman, municipality, rural)

There were also references to the care chains that arise in rural settings among women of different generations and formal female caregivers in families with dependents:

*“I’ve been at home and I’ve seen that my wife was there, three female formal caregivers were there, the two daughters were there, the sons spending the night, the whole world going crazy. We came here and everyone was so at home.”*

(D64, 2014, man, nursing home, rural)

Discussions regarding the fight for gender equality were more common in urban areas, with significant differences depending on the contributor’s sex. In the workplace, men referred to the de facto equality that exists nowadays, while women emphasized that there is still progress to be made. In the context of couples, emphasis was placed on the influence of women joining the workplace in terms of the fair distribution of caregiving tasks, and the freedom of modern women in terms of their love life:

*“Women used to work less than they do now. Today women are the same as men, at work and in everything, and of course they have to share: the housework, the children, everything. They have to share everything. […] in ten years, women will have overtaken men, won’t they? In everything: the way they talk, their intelligence, their daily chores, their work. Men are more… I mean, they work less, to put it simply.”*

(D3, 2008, man, seniors’ recreation center, urban)

*“There’s a pay gap, women know that from November… we’re working for free. There’s a glass ceiling, there’s no way to break it. And then there’s also… well, the type of work, occupational segregation.”*

(D50, 2020, woman, seniors’ recreation center, urban)

*“I’m not talking about being smart or anything. I’m saying that there’s equality, equality now between women and men. There wasn’t before, men gave the orders and that was it.”*

(D3, 2014, man, seniors’ recreation center, urban)

*“What I also think is that they’re short-handed in the family. I mean, the kids: the mum’s working and the dad’s working, the kids are alone and stuck in their room…. that didn’t happen before, before you’d play with them, tell them stories, sing with them, go in and out…. and now? They’re really adrift.”*

(D3, 2014, woman, seniors’ recreation center, urban)

*“I don’t think it’s any good for them to go to bed with one today and others tomorrow, but I don’t think it’s any good for a woman to have to put up with a man for so long either, like they put up with them before.”*

(D11, woman, nursing home, urban)

*“I think it’s a completely different society to then, because now the circumstances mean that both of us have to work, and if we’re both working, of course the chores have to be shared.”*

(D24, 2013, man, seniors’ recreation center, urban)

Finally, the issue of female looks and appearance was present in interviewee responses, whether with reference to themselves or to other women. Female interviewees had experienced oppression resulting from the strict dress code imposed on older women, or considered that certain rules should be followed in terms of how women of their age dressed:

*“Some of them are a bit carefree [others laugh]. I’m not keen on short skirts. And then they sit on the sofa and there you go! [other laugh]. […] They don’t cross their legs and they don’t look, as if they don’t see themselves… they make me want to come out sometimes and say: madam, adjust your skirt a little, please.”*

(D30, 2015, woman, seniors’ recreation center, urban)

*“For example, I’ve got a friend, a very kind “friend” [sarcastic emphasis], who tells me: “at your age, you should always be dressed in black.”*

(D30, 2015, woman, seniors’ recreation center, urban)

### 3.3. Residential Strategies

Residential setting was a matter that arose with some frequency in responses, with the main debate surrounding ageing at home or in nursing homes. Choosing to live in nursing homes is sometimes interpreted as a way of freeing children from the caregiving burdens that can arise in situations involving dependency, whether in rural or urban municipalities:

*“Those who don’t think about anything except themselves, that’s not for me. It was always clear for me, because before my husband died, and he’s been ten years in the ground, we were already saying ourselves that we weren’t going to ruin our kids’ lives. […] And you know, lots of things change, but you have to adapt and that’s that. And when the time came for me, it was a bit tough, because it’s true that you’re breaking away from everything that’s been your life, your kids, your grandchildren.”*

(D64, 2014, woman, nursing home, rural)

*“It’s been [clear] for me from the start, from how I saw my parents and my parents-in-law and others die… from burdening the kids. I think, and I’d ask governments to plan for us to be able to go to nursing homes where we’re not depending on the family and we can be looked after.”*

(D49, 2020, man, nursing home, urban)

Added to this is a recognition that having access to the social and healthcare resources that nursing homes offer is a positive in certain cases compared to continuing to live in the family home:

*“You know, the thing is that there’s more affection, more human warmth at home, more… the family is… that’s obvious, isn’t it? The thing is that in a nursing home you’ll definitely be much better looked after, there are doctors around the clock… there are healthcare specialists … there’s everything, you’ll have anything you need.”*

(D45, 2019, man, seniors’ recreation center, urban)

*“even if you’ve got someone giving you a hand at home, looking after you, you’re not looked after like you are in nursing homes.”*

(D54, 2021, woman, academic institution, urban)

However, ageing at home with support is the preferred option among those living in urban centres, since moving to a nursing home often means losing family and social relationships. Some interviewees commented that nursing homes risk not being able to offer the necessary affective care for their residents, and it was even noted that ageing at home with support could generally prove the more economical option for the State:

*“social care today is mainly targeted at that, at home help. I think it would be really useful to focus more on that point. Because if it’s real home care, at certain times or for meals or sanitary needs, intensifying the focus on that area of care would mean people wouldn’t have to be admitted to nursing homes or hospitals, wouldn’t it? And it would even be useful in financial terms, because it would be cheaper for the authorities.”*

(D5, 2009, woman, seniors’ recreation center, urban)

*“Now there are people who are really suffering, because even family members come along, leave them and ignore them. That’s what I think is wrong.”*

(D38, 2017, woman, nursing home, urban)

*“Affection, deep affection. It’s the only thing that saves an older person: being truly loved. If they really love you, you forget all your problems; but if they ignore you, you’re lost. I see it here all the time, here at the [nursing] facility. I’ve been here for five years and I see it. I see people who don’t get any visitors for the whole year and that’s really something, you know, it’s really sad.”*

(D38, 2017, man, nursing home, urban)

A nursing home is therefore not always the most desirable alternative. However, it might be the most feasible choice for the family for financial reasons, when the facility is public, or a highly burdensome one in the case of having to bear the cost of formal caregiving whether at home or in a private nursing home:

*“I think it’s fundamental to be at home, because the patient is living in their environment, they don’t have that degree of dislocation that they sometimes get when they go into a nursing home. You can tell [the lower level of dislocation from] the family warmth, whether we like it or not, but it means having plenty of financial resources that unfortunately we don’t currently have.”*

(D45, 2019, woman, seniors’ recreation center, urban)

Centres for older people represent useful resources in the fight against loneliness. They can offer older people understanding and the chance to be heard by their peer group:

*“being like you say, alone at home, because today we’ve got a bit of freedom. We have these older people’s centres, and whether you like it or not, people who are alone or who are married and have reached a certain age, they get up and say: “well, I’m off to a seniors’ recreation center”. Fortunately there are a lot of them, particularly in the communities, and look, you go there like we’re doing now, and instead of being sat at home on your own watching the television or reading, we’ve got some entertainment. We can say: “well, I’m off to this seniors’ recreation center” and some people do IT there, some do… thousands of things in the end, and we’re entertained for the whole day. So marginalized, marginalized… to a certain extent. That’s what I think.”*

(D5, 2009, man, seniors’ recreation center, urban)

*“It’s my second home, and the truth is that it’s been brilliant for me because I’m a bit lazy about going out. If I let it be, I wouldn’t go out because I can’t be bothered, I’m lazy, but this makes me go out every day because I’ve got an obligation, I have to come… I’ve got some great companions and we’ve always got things going on and we always have to be doing something.”*

(D16, 2011, woman, seniors’ recreation center, urban)

Finally, opinions were divided in the responses as to whether it is preferable to live in rural environments or in the city, with frequent references to the resources available in urban centres as exerting a stronger pull than the peacefulness offered by rural areas:

*“We all like the countryside, of course, but maybe here we’re not quite as alone. And we know that the kids […] don’t have time to deal with older people in that sense, maybe we’re a bit selfish, and at least here they can come and do activities, talk to each other, tell each other what’s going on with them and have a bit more company than [clicking tongue] if they each live in a house in the countryside.”*

(D34, 2016, woman, seniors’ recreation center, urban)

*“Older people have many more distractions in the cities, yes, that’s definitely true. They also have the advantage of lots of hospitals and care services, which is more complicated in the countryside […] the countryside, well, you can switch off, and I think that it could also do them good to spend some time in the country… but in the city I find that they, I don’t know, it seems to be better for them, to fit them better.”*

(D34, 2016, woman, seniors’ recreation center, urban)

*“You get up in the morning and yes, the countryside is really beautiful, the little birdies singing and everything, but you’re alone. So the neighbours get on with their own business and you’re alone. I found it lonely and so did my husband.”*

(D34, 2016, woman, seniors’ recreation center, urban)

## 4. Discussion

This article investigates older people’s social self-image through a qualitative content analysis of their statements regarding matters of interest. Opinions were taken from those offered on the Radio Nacional de España program, Together, Step by Step (Juntos Paso a Paso). The results are divided into three areas: the representation of older people in the media, gendered ageism and residential strategies. All three themes highlighted in the previous section affect male and female experiences with ageing. The representation of older people in the media reinforces ageist stereotypes, which leads to the exclusion of older people from the community at both a societal level [10] and through self-exclusion [11] impairing active ageing by limiting participation.

Participation is also limited by gender. As older women are the principal caregivers of the family unit, they may be unable to engage in full participation, impacting their quality of life. Finally, residential strategies shapes both active ageing and quality of life [12]. In conclusion, all three areas are relevant to active ageing since they play an essential role in quality of life, and especially in the later stages of life.

### 4.1. Positive and Negative Images of Older People in the Media

From the responses collected, program participants clearly felt that older people were under-represented in the media, the entertainment industry, and social media. Along the same lines, previous studies on media images of old age report that older people are an under-represented group and that they are often subject to stereotypical representations [27,43]. These stereotypes contribute to reinforcing a homogeneous and ageist view of old age based on decline, vulnerability, and fragility by linking older people to three main topics: problems with health, sexuality, and death [44]. Evidence of this is found in the striking prevalence of disability-related content from 2008 and particularly 2009, the early years of the radio program. The basis for this fact might also be the publication by the Spanish National Institute of Statistics (INE) of a macro-survey on disability, personal autonomy and situations of dependency in 2008 [45]. This represented a significant update to the previous “Survey on Disabilities, Impairments and State of Health” from 1999.

This tendency to portray an image of vulnerability and homogeneity when representing old age appears to have intensified in recent years with news regarding the COVID-19 pandemic, indicating that this stereotyping remains a latent issue today [23,24,46,47,48].

Recent studies have also noted the emergence of another media profile that is more related to successful ageing [44]. This is based on other pre-existing conceptions [49] and positive stereotypes regarding older people, such as that they are friendly but incompetent [50]. However, it is also noteworthy that this conception places a large part of the burden in terms of ageing well on older people themselves, again exemplifying a single, homogeneous view of how one should age satisfactorily [44]. Both negative attitudes and those that imply some kind of positivity therefore may have adverse impacts.

The media and entertainment industries act as a mechanism for the transmission and reinforcement of entrenched beliefs in society, regardless of the extent to which those beliefs reflect reality, are based on stereotypes or simplifications of social reality, or are constructed based on proven scientific evidence. When examining the image of older people in the media, it appears important to refer not only to the sources in which older people play a passive or secondary role but also to those areas where they are active participants, which has been the study aim in this work.

### 4.2. Gendered Ageism and the Role of Old People in Their Families and Society

The most commonly cited forms of age-based discrimination by interviewees concerned the consequences suffered as a result of the invisibilization of older people. Participants in the radio programs analysed in the study explained their opinions on three issues with respect to this form of discrimination: caregiving work for their families, older people’s rights to continue contact with grandchildren in cases of parental divorce, and barriers between grandparents and grandchildren as a result of differing digital habits.

It is significant that these interviewees’ experiences of invisibilization were closely related to family relationships. Caregiving tasks were one of the most commonly mentioned aspects in the statements analysed, with interviewees describing the often-undervalued contributions that they made to families by caring for the grandchildren. These contributions benefit parents by lightening their burden, but they also help society as a whole due to the economic benefits generated by enabling both parents to remain in full-time work.

Since the entry of women into the remunerated workforce started to become widespread in Spain, particularly from the latter part of the twentieth century, it has been observed that the use of time has transformed considerably for females but not so much for males. In other words, although family units began to share financial obligations, household and caregiving duties remain an area in which there is a long way to go in terms of integrating men [51,52].

Caregiving tasks are closely linked to gender roles. In previous research, both in Spain [53] and in other countries [54], it has been notable that caregiving in relation to grandchildren is mainly carried out by grandmothers, particularly in terms of activities requiring emotional support and physical care, while grandfathers are more dedicated to supporting leisure activities.

With respect to age, stereotyping and discrimination defined by gender is referred to as gendered ageism. The WHO has recently defined this term as “the differences in ageism faced by women compared with men” [7]. In other words, gendered ageism refers to the influence that gender roles have on beliefs and attitudes regarding old age and coping with ageist situations [16].

This intersection between age and gender does not suddenly appear in old age. Rather, as with many other characteristics, its origins go back to the start of the life course, and it is maintained during ageing [55]. Gender roles mean that women and men do not age under the same conditions or with the same opportunities; they also affect the formation of different, specific sex-based stereotypes and prejudices.

This idea has been adopted by the active ageing paradigm since it was established with the life course approach [1], which is widely recognized in ageing-related research: the opportunities, obstacles, events and milestones occurring in a person’s life have an influence over the rest of their life. Therefore, the existence of different areas of oppression gives rise to disadvantages in an individual’s life and environment: these areas include gender, socioeconomic status and ethnicity, among others [56].

From childhood onward, women are assigned as the primary carer of the home. They maintain this status in old age for reasons including the association of caregiving tasks with femininity, which ends up justifying the tendency to believe that they are better at performing these tasks, as some authors have noted [54,57]. Caregiving responsibilities hence represent work that women themselves end up normalizing.

In view of the responses analysed in this study, women’s complaints are not based solely on an unfair burden, as is the case among men. They are more focused on the restrictions caused in terms of their use of time due to obligations to care for grandchildren, whether externally imposed or self-imposed due to social norms. These complaints are not isolated. Other studies have also identified the greater involvement of older women in caregiving activities related to their grandchildren, which have a particular consequence of limiting their social and political participation in activities that require greater time and effort [57]. Therefore, since participation is one of the main pillars of active ageing, their citizenship becomes limited from being fully exercised.

On a different matter, interviewees also referred to the consequences of their children’s divorces in terms of relationships with their grandchildren. Their view was that it is necessary to take into account the rights of grandparents—and, one might add, grandchildren—to remain in contact despite parental divorce.

In the present day, increased longevity and life expectancy has meant that the oldest and youngest generations are living together for more time. This requires increased interest in developing the relationships between these generations and safeguarding their rights. Social research into the functioning of family systems has generally involved studies of nuclear family sub-systems: relationships between and among parents and siblings [58]. However, other perspectives should also be introduced that also affect the relationship between the various sub-systems, such as the oldest generations of the family.

In processes of family crisis, which would include divorce, there are two lines of action that particularly affect grandparents [59]. One is their assumption of a “family watchdog” role, providing stability to the children during the separation process by increasing their caregiving time and responsibilities [60]. Another is their loss of contact with grandchildren. This generally has more impact on the paternal grandparents for reasons such as change of residence following parental separation and unequal custodial regimes that favour one parent or the other [60,61]. In this respect, it should not be ignored that the influence of the link between caregiving roles and femininity and women plays a significant role in the contact between grandparents and grandchildren. This means that maternal families are an ongoing resource for mothers in divorce processes and grandparents from maternal families are hence more involved in caring for their grandchildren than those from the paternal family [59].

Another significant aspect in the interview responses was the use of information and communication technologies (ITCs) and the digital gap that exists between the older people interviewed and the younger members of their environs, especially their grandchildren. Interviewees argued that the mass use of new technologies creates a barrier that hinders interaction, meaning that the time they spend together is not shared and there is none of the communication there would be in the absence of electronic devices.

There is an age-based digital gap in Spanish society. Though it is gradually closing, it remains particularly prevalent among the most advanced ages (75 years and above). In recent years, following the onset of the pandemic and the experience of lockdown, the Spanish population aged over 65 years has increased its day-to-day internet use, with 56.3% of people aged 65 to 74 using the internet daily in 2021 (compared to 43.3% in 2019). However, this figure plunges to 25.9% among people aged 75 to 84 and 8.7% among those aged 85 and above in 2021 [62].

This context hence reflects the varying digital habits of the different generations, hindering communication between grandparents and grandchildren. From the perspective of active ageing and ageism, digital literacy has become a key issue to ensure that older people participate in society. Notable studies in this regard include the research published by Alonso Ruiz, *et al.* [63], who stressed the usefulness of digital leisure activities in increasing wellbeing among older people by expanding communication with loved ones, particularly during lockdown, and the opportunities for intergenerational connection offered by new technologies in terms of offering a learning process whereby the younger people can teach the older ones.

Two ageism-related aspects affect the integration and use of technologies by older people. Although they were not referred to in the statements analysed, these two aspects are closely linked to age-based discrimination and older people. First, digital devices, social media and other forms of digital communication and leisure are not designed taking into account the needs of older people, which affects the user experience [64,65]. This is especially important when one takes into account that a large number of basic services have become digital in the recent years, including banking, healthcare [66], and taxation, with local services relegated to a secondary and ill-resourced position.

Second, ageist beliefs influence older people to learn digital skills: this implies a risk that the older people who have not had an opportunity to integrate in terms of ITC use since the start of the digital transformation—those in the highest age bands—will assume socially established ageist stereotypes that make them less willing to incorporate new technologies and electronic devices into their lives [67].

Other expressions of gendered ageism arising in the interviewee responses that have been extensively covered in previous studies relate to aesthetics and the regulation of the female body at any age [16,68,69,70,71,72]. For the older female interviewees, this entailed the standardization of aspects such as their appearance and clothing through defined rules applicable to the person as a woman and an older adult.

The body undergoes physical changes over a lifetime, producing age-related signs that materialise the passage of time. These changes encourage a form of gendered ageism that particularly affects older women, given that standards of female beauty are related to youth, even though unrealistic beauty standards prevail at all ages. In contrast, men do not always face this issue, since in their case certain age-related physical features can be associated with attractiveness. Women are therefore driven to decide between reinforcing the authenticity of their image as older women, for example by choosing grey as opposed to dyed hair, or fostering the dominance of youthful imagery by concealing the signs of age [69,72].

Female interviewees described how aesthetic rules and appearance norms affect how they dress. Responses noted the expectations for widows—mainly older women—to use specific colors or styles of clothing from a certain age, particularly involving hiding the body. This is all closely linked to the image projected of women in society, which as Veresiu and Parmentier [73] note is profoundly influenced by the fashion and beauty industries and advertising. Women and young bodies are protagonists in advertising and fashion, with images of older women rarely featuring in campaigns. Women of a certain age are hence relegated to a secondary position, assuming that there is no reason to attempt to stand out by dressing in a particular way or that in order to do so they would have to adhere to youth-related standards of beauty.

Twigg [74] affirms that fashion trends tend to be initially adopted by young age groups and then extended to the older groups. This indicates the process that older women have to follow to be integrated into society, whereby they are encouraged to constantly hide the signs of age through their aesthetic expression.

Finally, there were notable statements regarding the pursuit of gender equality, although this only occurred in the urban context. Interviewees referred to the evolution of opportunities and gender roles in recent decades with respect to the allocation of household chores, and particularly with regard to women joining the paid workforce. In addition to this, interviewees who talked about their past jobs described the occupational segregation they had faced, including dedication to “their work” (housewife) and including driving. Of note on this point were references to the persistent gender gap, not only in the job market but also in the performance of household chores that are not classified as paid work.

Research into equality and feminism among older people is difficult to find; it is almost always included in publications dedicated to gender violence. For example, von Humboldt *et al.* [75] conducted qualitative research with 173 participants aimed at analysing the perspectives of bullying in old age. They reported several female interviewees who identified the gender-based discrimination they suffered as older women and one of them explained the situations of violence to which she was exposed.

It is only in recent years that interest has arisen in Spain regarding the study of older women’s positions with respect to gender violence [76,77,78,79] on one hand, and gender violence in rural settings on the other hand [80].

This geographical division in the statement might be largely due to the normalization of feminist discourse in the urban environment in contrast to rural settings, although it has not proven possible to find references in support of this argument.

### 4.3. Ageing in Place or Growing Older at Home vs. Residential Care

Several global action frameworks for active and healthy ageing advocate adapting residential environments to make them accessible, safe, inclusive, and sustainable, particularly for older people and other vulnerable groups [3,81,82]. However, people also take decisions during their lifetimes regarding their residential environment, housing and the community (rural or urban) in which they want to live during old age. These strategies are related to their residential desires in terms of maintaining or improving quality of life. They will be affected by levels of health-related and functional competence and personal circumstances [83], family and support network, and personal and financial resources available to support ageing in the person’s preferred environment [5,84].

Recent studies based on the perceptions and preference of older people indicate that ageing in place is linked to a feeling of belonging or connection to a place and perceptions of security, familiarity, independence, and autonomy. This applies to both housing and the wider community [85,86,87,88]. The responses analysed show that both people living at home and those in nursing homes emphasized a preference for age-friendly environments that would permit growing older at home with access to home care or support services.

Growing older at home appears preferable when older people enjoy or expect to enjoy good health. Meanwhile, structural shortfalls in the provision of formal support, in addition to other sociodemographic, psychological, and attitudinal factors, may explain the reduced desirability of ageing at home where people are more fragile, with a preference to move into the homes of other family members that can provide care [89]. However, the statements revealed older people specifically rejecting the idea that they might be a burden on their family, and particularly for their children. This image of being a burden caused preferences to shift to living in a nursing home where necessary, whether due to the availability of more social and healthcare resources or because financial factors made remaining at home a less affordable alternative than institutional care. A recent literature review notes that older people prefer to receive care in their own physical and social setting when they have moderate needs but opt for the provision of long-term residential care when their needs are greater [90].

These statements coincide with those obtained from other research into active ageing in Spain, which underline the interest of older people in choosing a residential context that is different from their home when they want to avoid concerning their children [91], that provides security in terms of healthcare and attention [85], and that has no obstacles and is suited to their physical and mental needs [87]. In financial terms, owning a property tends to result in remaining in place, while not being a homeowner can be a factor that forces people to move into nursing homes [85,86,87].

However, nursing homes for older people have been seen as places where family and social relationships are lost. This was essentially observed in responses provided by people who were already institutionalized. Added to this is the experience of living in nursing homes during the COVID-19 pandemic, which gave rise to a profound reflection regarding the need to review the current model to improve the age-friendliness and security of nursing homes and make them environments for active and healthy ageing [92,93,94].

There have also been accounts from older people of viewing themselves as a burden and hence fearing deterioration in their family relationships if they have to move in with children or other relatives [95]. Deterioration or loss of family relationships is part of the experience of being alone in old age that can make a difference when choosing residential strategy. While this may sometimes be synonymous with autonomy (living alone), on other occasions, when the solitude is imposed and not voluntary, it may result in chronic and severe negative feelings with an adverse impact on health and wellbeing [91]. In some cases, the use of recreation centers for older people by those ageing at home can be considered a resource to combat loneliness, by encouraging participation and involvement in activities [12,96]. In other cases, living in a nursing home can contribute to alleviating loneliness, when the older people in those facilities are satisfied with their residential setting and with the other residents. This can increase feelings of belonging [91].

In terms of the friendliness of residential settings for ageing in place, responses from interviewees living in urban environments involved the benefits of living in an urban or rural setting. These interviewees expressed a preference for the former, highlighting advantages such as the presence of social and healthcare resources to encourage older people to participate in activities and interact with other persons, thereby preventing loneliness and facilitating access to healthcare. However, studies on ageing in rural settings have reported positively on cognitive health and social support [97], intergenerational social contact [98], and increased integration into society and with neighbors in these smaller areas. Said matter is also related to security in the residential setting [87]. However, these studies have also identified as a negative aspect of rural settings the difficulty of accessing health services [87], particularly for disabled populations [99], which include older people in situations involving dependency [79]. In the context of the COVID-19 pandemic, it remains unclear whether rural settings are healthier environments or if there may be less likelihood of catching the virus in these areas [79]. However, other problems have been highlighted in rural settings during the pandemic, including poor internet connectivity and the digital gap. This has had an impact by causing inequality of medical care for older people [100], loneliness, isolation, and other problems caused by social distancing [101].

### 4.4. Study Limitations

Limitations were encountered in the course of this research that affected its development, particularly in terms of obtaining an adequate level of inter-coder agreement (ICA). After calculating the ICA, the research team met to discuss the causes of this issue and reached various conclusions.

First, it was noted that the nature of the matter subject to analysis might have contributed to obtaining a lower level of agreement due to its content: a secondary source not designed for qualitative analysis made homogeneous codification difficult. The documents analysed came from a concise source of information: segments of a radio program that were each approximately five minutes long. Although these segments had a defined structure involving the presenting team and the interviewees, presenter interjections were frequent (albeit added post-interview, as the program is not broadcast live) and the content of interviewee responses was commonly restricted to a few sentences without the possibility of going into greater depth. This may have represented an obstacle to producing a single interpretation of the data. Second, the absence of prior agreement as to the length of quotations, as well as the continuous appearance of text that was not relevant to the analysis—like technical details and presenter interjections—contributed to diminishing the quantification of agreement.

In short, following the debate regarding calculation of ICA, the team identified that the figure obtained was based less on a disparity in the use of the established codes and more on causes linked to the analysed subject matter not identified prior to coding. However, although ICA did not reach a satisfactory statistical level, it was not applied to obtain a specific coefficient for validity but in an attempt by the research team to create a study that would be reliable and replicable by other teams, as other authors have previously noted [102]. The qualitative research forming the basis of this study is based on the research team underpinning its interpretations with the shared conceptual frameworks to which the study adheres. This intention was present from the outset of the research, as shown by the use of two widely recognized conceptual frameworks in social research on ageing and old age: the pillars and determinants of active ageing and the dimensions of ageism established by the WHO [7]—stereotypes, prejudice and discrimination.

The distribution of content for the Together, Step by Step radio program also reflected a dominance by the population of the Community of Madrid. This may be explained by the geographical location of the program’s headquarters: the city of Madrid. The random sampling of documents also revealed a scarcity of content from rural settings, which meant a decision was made to include those documents in a controlled manner to ensure that they were sufficiently represented.

### 4.5. Future Lines of Research

A novel aspect of this study was the use of a media-based information source (a radio program) involving active participation by older people. Although radio is not currently a key media source for media content analysis compared to television, the internet, or social media, it remains widely used among the Spanish population. This adds relevance to social research regarding the content broadcast over radio. The selection of this study source and its stability over time made it possible to obtain statements from older people spanning a thirteen-year period regarding aspects that are significant for ageing and age-related conceptions.

Future research could extend the use of this type of information source, which may prove highly useful in terms of examining the evolution of older people’s statements over time. It is also a source of intuitive and non-professional participation and analysis of the study group.

It is also necessary to continue researching the relationship between ageism and gender roles and their impact on past, current, and future generations of older people in terms of how those people perceive their own ageing compared to established beliefs. It would therefore be useful to examine the particular age-related matters that have arisen in relation to gender, such as the relationship between old age and aesthetic expression and appearance for older women, or other matters not addressed in this study but closely linked to gender stereotypes, such as sexuality.

Finally, taking into account the medium and long-term impacts of the COVID-19 pandemic, in coming years, it would be useful to examine potential changes in perspectives regarding life and old age as a result of the political, economic and social consequences of the health crisis by conducting comparative pre- and post-pandemic studies. Along similar lines, climate change represents a global challenge with local implications that should be addressed from a gender perspective to contribute to reducing vulnerability and encouraging climate change adaptation among older women in urban and rural settings [103,104].

## 5. Conclusions

The aim of this study was to research older people’s social self-image by examining statements broadcast on a radio program with a target audience of older people.

Its thirteen-year timespan and diversity of topics means that the Together, Step by Step (Juntos Paso a Paso) (RNE) sound archive can be considered a viable secondary source to analyze the evolution of older people’s social self-image. It is also a fact that there is a scarcity of active participation by older people in the media. This increases the interest in studying this kind of source, where older people express their opinions frequently and based on experience. Participant responses were analysed using the active ageing framework proposed by the WHO as reinforced by subsequent paradigms (age-friendly cities and communities, ageism, etc.): this tool reinforces the heterogeneity of forms of ageing and, as a result, the fight against a negative image of ageing and old age.

Three lines of research were pursued when analyzing the sound archive: ageism, gender and ageing in place.

First of all, it was found that the more frequent manifestations of ageism among the program participants were implicit; while not explicitly ageist, they reiterated and consolidated stereotypes and prejudices regarding ageing and old age. There was also a close connection to the invisibilization of the group in the contexts of family, society and the media. The social representation of old age in the media was particularly referred to in urban contexts (>10,000 inhabitants), where an image was constructed of older people as passive, disposable subjects. This tendency has intensified during the pandemic, as noted by other authors. In contrast, there were also positive stereotypes of old age (wisdom; experience) and a positive assessment of available resources such as seniors’ recreation centers, which are potential agents for the promotion of active ageing. The dissemination of this profile in the media can significantly change the perception of old age and the role of older people in society, although there is a need to remain alert and avoid falling back into homogeneous stereotyping about how to age well.

Interviewees linked old age to the expectations older people faced in the family context (generally from their children) in terms of taking care of grandchildren. Differences were observed in the construction of caregiving depending on the environment. While older people in rural areas (<10,000 inhabitants) stressed emotional value and the crucial role of caregiving in maintaining quality of life for their children and grandchildren, older people in urban areas noted that caregiving was also a burden for them, like any work and regardless of gender. This burden was described as an opportunity cost given that caregiving requires time that could be used for other activities that are more satisfactory for older people, enabling them to increase their social participation and engage in other leisure activities that would promote active ageing and quality of life.

In relation to the second line of analysis (gendered ageism), constructions and attitudes surrounding caregiving were precisely the area featuring the most pronounced gender-related differences in the statements. For older women, taking care of the grandchildren (a task that rural residents expressed more willingness to perform) played an almost-central role in their family identity during old age, and caregiving extended to their partners. In the case of dependent family members, caregiving seemed to be extended to the younger female members of the family, creating a caregiving chain.

This caregiving has had an impact in terms of differences between male and female time use throughout the life cycle; in their statements, occupational segregation, for example, has been mentioned to have a particular impact in rural settings. This division involved men taking care of productive work and women handling reproductive work. Post-retirement, men have continued to occupy the public sphere through leisure and women have remained the main protagonists with regard to the household and associated chores.

Discussions regarding the evolution of these roles and specifically concerning gender equality were more common in urban areas, with gender-based differences. In general, women have a positive view of progress in gender equality, while men fear the threat to their status. This is a matter of interest for future examinations of the intersection between age and gender, taking into account its extreme scarcity in the scientific literature.

In the case of women, particular references were made to self-imposed and societal expectations regarding dressing or behaving in a way that is expected of older women. This has been extensively studied by the scientific community in relation to gendered ageism. The anti-ageing pattern expressed through fashion that is mainly targeted at younger women has been noted as contrasting with an acceptance of the authenticity of older women through the reinforcement of the features characteristic of their gender and age.

Both rural and urban statements contained stereotyped beliefs about young people and their current lifestyles, which are seen as different from the interviewees’ youths. This perceived gap, related to the digital gap between younger and older people, might hinder the development of intergenerational relationships. However, some authors have affirmed the potential of intergenerational collaboration through digital media. Moreover, the literature has emphasized the benefits of digital literacy among older people in the sense that it offers them more avenues for communicating with loved ones. However, the process of digital integration for older people must take into account the influence of ageist beliefs that older people themselves have assumed and which hinder their use of technology, in addition to the difficulties caused by electronic devices and virtual environments that have not been designed with the needs of older people in mind.

With regard to intergenerational relationships, another cause for complaint is the secondary position of grandparents with respect to contact with their grandchildren in processes involving parental divorce. Various authors have noted two potential tendencies in terms of relationships with grandchildren in this regard. First, post-separation geographical relocation and custodial regimes cause distancing and do not take into account the rights of grandparents and grandchildren to remain in contact. Second, the absence of two parents to take care of the children can intensify the caregiving role assumed by older people, who become a source of stability for their grandchildren during divorce processes.

Finally, in relation to the last line of analysis (ageing in place), the decision regarding whether to grow older at home or in a nursing home is affected by internalized ageism among older people. Nursing homes are seen as a resource to avoid or alleviate the caregiving burden that children might face, whether in a rural or urban context.

Although the healthcare resources offered by nursing homes are viewed positively and public nursing homes may be more economical than other alternatives, they are not considered the most attractive option. People living in urban environments prefer home care, since moving to a nursing home would entail distancing from their neighborhood and family network. Recent studies in this regard have been unanimous: older people prefer to remain in their physical and social environment compared to a nursing home for as long as they have moderate care needs, although they accept the move to a nursing home when their needs are greater.

Seniors’ recreation centers represent another noteworthy alternative for preventing undesired solitude and developing social networks. Participants found urban contexts preferable and more age-friendly, given the increased levels of available resources including hospitals and public transport. Moreover, urban residential settings have a higher population density. Interviewees felt that this made it easier to create neighbourhood networks and hence offered them more opportunities to active ageing with a good quality of life.

## Figures and Tables

**Table 1:** Distribution of interviews in “wise words” segment by size of municipality.

Size of Municipality	No. of Programmes Analysed (and % of N in Each Size)	N = Total No. of Programmes (2008–2021)
Less than 10,000 inhabitants	11	(100%)
10,000 to 49,999 inhabitants	3	(9.1%)
50,000 to 499,999 inhabitants	15	(13.9%)
More than 500,000 inhabitants	36	(10.1%)
Various ^1^	1	(10.0%)
Size unknown ^2^	-	-
**TOTAL**	**66**	**(12.2%)**

^1^ Programme specifies various locations. ^2^ Programme does not specify a particular location.

**Table 2:** Distribution of interviews in “wise words” segment by residential context.

Residential Context	Description	No. of Programs Analysed (and % of N in Each Context)		N = Total No. of Programs (2008–2021)
Seniors’ recreation centers	Interviews with older people attending centers for older people.	32	(11.6%)	277
Nursing homes	Interviews with older people living in nursing homes.	22	(14.8%)	149
Day care centers for elderly people	Interviews with older people attending day care centers.	4	(10.8%)	37
Municipalities	Interviews conducted in public in municipalities, without specifying whether location is a specific resource for older people.	4	(50.0%)	8
Academic institutions	Interviews with older people in the university context (mature studies)	2	(28.6%)	7
Foundations, Federations	Interviews with older people at Spanish foundations and federations.	2	(25.0%)	8
Other (authorities, companies, associations)	Interviews with older people in other contexts.	-	-	56
**TOTAL**		**66**	**(12.2%)**	**542**

**Table 3:** Citation distribution for most commonly used codes ^1^ in codification based on municipality size.

	Citations in Municipalities with Fewer Than 10,000 Inhabitants		Citations in Municipalities with More Than 10,000 Inhabitants		Totals	
Concept of Active Ageing	**-**	**0.0%**	26	3.1%	26	2.6%
Quality of life	-	0.0%	31	3.7%	31	3.1%
Pillar: Security	-	0.0%	56	6.6%	56	5.7%
Pillar: Participation	7	4.9%	47	5.6%	54	5.5%
Pillar: Health	2	1.4%	44	5.2%	46	4.7%
Transversal determinant (gender)	30	21.1%	52	6.2%	82	8.3%
Social determinants (family network)	23	16.2%	69	8.2%	92	9.3%
Social determinants (caregiving)	16	11.3%	34	4.0%	50	5.1%
Social determinants (social network)	5	3.5%	27	3.2%	32	3.2%
Services determinants (health and social services)	4	2.8%	33	3.9%	37	3.8%
Economic determinants (socioeconomic status)	10	7.0%	17	2.0%	27	2.7%
Physical environment determinants	1	0.7%	21	2.5%	22	2.2%
Behavioural determinants (habits)	-	0.0%	29	3.4%	29	2.9%
Personal determinants (psychological factors)	2	1.4%	18	2.1%	20	2.0%
Ageism: implicit	2	1.4%	66	7.8%	68	6.9%
Ageism: invisibilization	5	3.5%	32	3.8%	37	3.8%
Ageism: loneliness	1	0.7%	23	2.7%	24	2.4%
Ageism: burden	3	2.1%	6	0.7%	9	0.9%
Ageism: self-image (personal development)	1	0.7%	28	3.3%	29	2.9%
Ageism: positive self-image	-	0.0%	24	2.8%	24	2.4%
Ageism: positive prejudices	3	2.1%	15	1.8%	18	1.8%
Newly created: Active Ageing promotion programs	3	2.1%	-	0.0%	3	0.3%
Newly created: Use of time	10	7.0%	31	3.7%	41	4.2%
Newly created: Implicit sexism	-	0.0%	22	2.6%	22	2.2%
Newly created: Generational differences	12	8.5%	18	2.1%	30	3.0%
**TOTALS**	**142**	**100%**	**843**	**100%**	**985**	**100%**

^1^ Only codes with at least 2% of citations in each size of municipality are presented. As such, the “Totals” row corresponds to the total project citations.

## Data Availability

The data used in this research can be accessed in audio format through this link: https://www.rtve.es/play/audios/juntos-paso-a-paso/ (accessed on 13 April 2022).
